# The Cell Signaling Adaptor Protein EPS-8 Is Essential for *C. elegans* Epidermal Elongation and Interacts with the Ankyrin Repeat Protein VAB-19

**DOI:** 10.1371/journal.pone.0003346

**Published:** 2008-10-03

**Authors:** Mei Ding, Ryan S. King, Emily C. Berry, Ying Wang, Jeff Hardin, Andrew D. Chisholm

**Affiliations:** 1 Department of Molecular, Cellular and Developmental Biology, University of California Santa Cruz, Santa Cruz, California, United States of America; 2 Cellular and Molecular Biology Graduate Program, Department of Zoology, University of Wisconsin, Madison, Wisconsin, United States of America; 3 Division of Biological Sciences, University of California San Diego, La Jolla, California, United States of America; 4 Department of Zoology, University of Wisconsin, Madison, Wisconsin, United States of America; University of Giessen Lung Center, Germany

## Abstract

**Background:**

The epidermal cells of the *C. elegans* embryo undergo coordinated cell shape changes that result in the morphogenetic process of elongation. The cytoskeletal ankyrin repeat protein VAB-19 is required for cell shape changes and localizes to cell-matrix attachment structures. The molecular functions of VAB-19 in this process are obscure, as no previous interactors for VAB-19 have been described.

**Methodology/Principal Findings:**

In screens for VAB-19 binding proteins we identified the signaling adaptor EPS-8. Within *C. elegans* epidermal cells, EPS-8 and VAB-19 colocalize at cell-matrix attachment structures. The central domain of EPS-8 is necessary and sufficient for its interaction with VAB-19. *eps-8* null mutants, like *vab-19* mutants, are defective in epidermal elongation and in epidermal-muscle attachment. The *eps-8* locus encodes two isoforms, EPS-8A and EPS-8B, that appear to act redundantly in epidermal elongation. The function of EPS-8 in epidermal development involves its N-terminal PTB and central domains, and is independent of its C-terminal SH3 and actin-binding domains. VAB-19 appears to act earlier in the biogenesis of attachment structures and may recruit EPS-8 to these structures.

**Conclusions/Significance:**

EPS-8 and VAB-19 define a novel pathway acting at cell-matrix attachments to regulate epithelial cell shape. This is the first report of a role for EPS-8 proteins in cell-matrix attachments. The existence of EPS-8B-like isoforms in Drosophila suggests this function of EPS-8 proteins could be conserved among other organisms.

## Introduction

Many organisms and organs develop from spherical or ovoid primordia that undergo elongation along an axis. The cellular bases for these elongation movements have been explored in a number of organisms [1]. In organs such as the vertebrate notochord, internally generated hydraulic pressure is resisted by an extracellular sheath whose geometry distributes the forces to the ends of the cylinder, resulting in elongation of the organ. The elongation of the *Caenorhabditis elegans* embryo is another example of a pressure-driven elongation process, but differs from the notochord in that the forces are generated by precisely arranged circumferential bundles of actin filaments in the apical layer of the enveloping epidermis. Contraction of these circumferentially oriented actin bundles (CFBs) in the epidermis causes coordinated contraction of the epidermal cells, and concomitant elongation of the epidermal cells (and all internal tissues) along the perpendicular anterior-posterior axis [2].

Analysis of elongation defective mutants has identified epidermal proteins that regulate actin-based contractions, supporting the model that actomyosin based contraction of lateral epidermal cells provides the driving force for epidermal cell shape change [3]–[6]. Epidermal elongation also requires epidermal cell-matrix attachment structures known as fibrous organelles [7] or trans-epidermal attachments (TEAs) [8]. Trans-epidermal attachments are composed of apical and basal hemidesmosome-like membrane plaques connected by intermediate filaments (IFs). TEAs are found in dorsal and ventral epidermal cells, where they connect underlying body wall muscles (or their associated basement membrane) to the apical cuticle. Within epidermal cells, TEAs are further segregated into stripes between the circumferential actin bundles.

Several components of attachment structures are essential for epidermal elongation. VAB-10A, the *C. elegans* ortholog of the cytoskeletal linker protein Plectin, is localized to the membrane-proximal plaques of TEAs, and is essential for elongation [9]. The cytoplasmic intermediate filament proteins IFB-1 and IFA-3 are also found in embryonic attachment structures and are required for elongation [10]. The ankyrin repeat protein VAB-19 is also essential for epidermal elongation [11]. VAB-19 localizes to trans-epidermal attachments but is not required for their initial assembly. VAB-19 instead appears to be required for later localization of attachment structures to muscle-adjacent regions of epidermis.

VAB-19 is a member of a conserved family of ankyrin repeat containing proteins thought to function in the actin cytoskeleton [12]. A human ortholog of VAB-19, Kank, was identified as a candidate tumor suppressor for renal cell carcinoma [13]. In mammalian cells the Kank protein can inhibit actin stress fiber formation via the RhoA GTPase [14]. Kank is thought to regulate RhoA as part of a complex with 14-3-3 proteins, which interact with coiled coil motifs in the Kank N-terminus. However, many questions remain concerning how VAB-19/Kank related proteins are regulated and how they function in vivo. To understand how VAB-19 functions in *C. elegans* epithelial cell shape change we focused on its most highly conserved region, the ANK repeat domain, which is necessary for VAB-19 function but not for its subcellular localization [11]. As ANK repeats function as protein-protein interaction interfaces, VAB-19 may bind one or more conserved proteins via this domain.

Here we report that the cell signaling adaptor protein EPS-8 interacts with the VAB-19 ANK domain, and that EPS-8 colocalizes with VAB-19 at epidermal attachment structures. Eps8 has been implicated in many aspects of growth factor signaling and regulation of the actin cytoskeleton [15]. Vertebrate genomes encode multiple Eps8-related genes [16], whereas the *C. elegans* genome contains a single locus, *eps-8*. Previous work has showed that *C. elegans* EPS-8 functions in regulation of the apical actin cytoskeleton of the intestinal epithelium [17] and in promoting basolateral localization of the epidermal growth factor receptor ortholog LET-23 in vulval epithelial cells [18]. We show here that EPS-8 also has an essential role in the morphogenesis of the embryonic epidermal epithelium, apparently independent of a direct interaction with the actin cytoskeleton. Our studies reveal a novel role for a member of the Eps8 family in epithelial cell-matrix attachments.

## Results

### EPS-8 and VAB-19 interact directly and colocalize to trans-epidermal attachment structures

**Figure 1:**
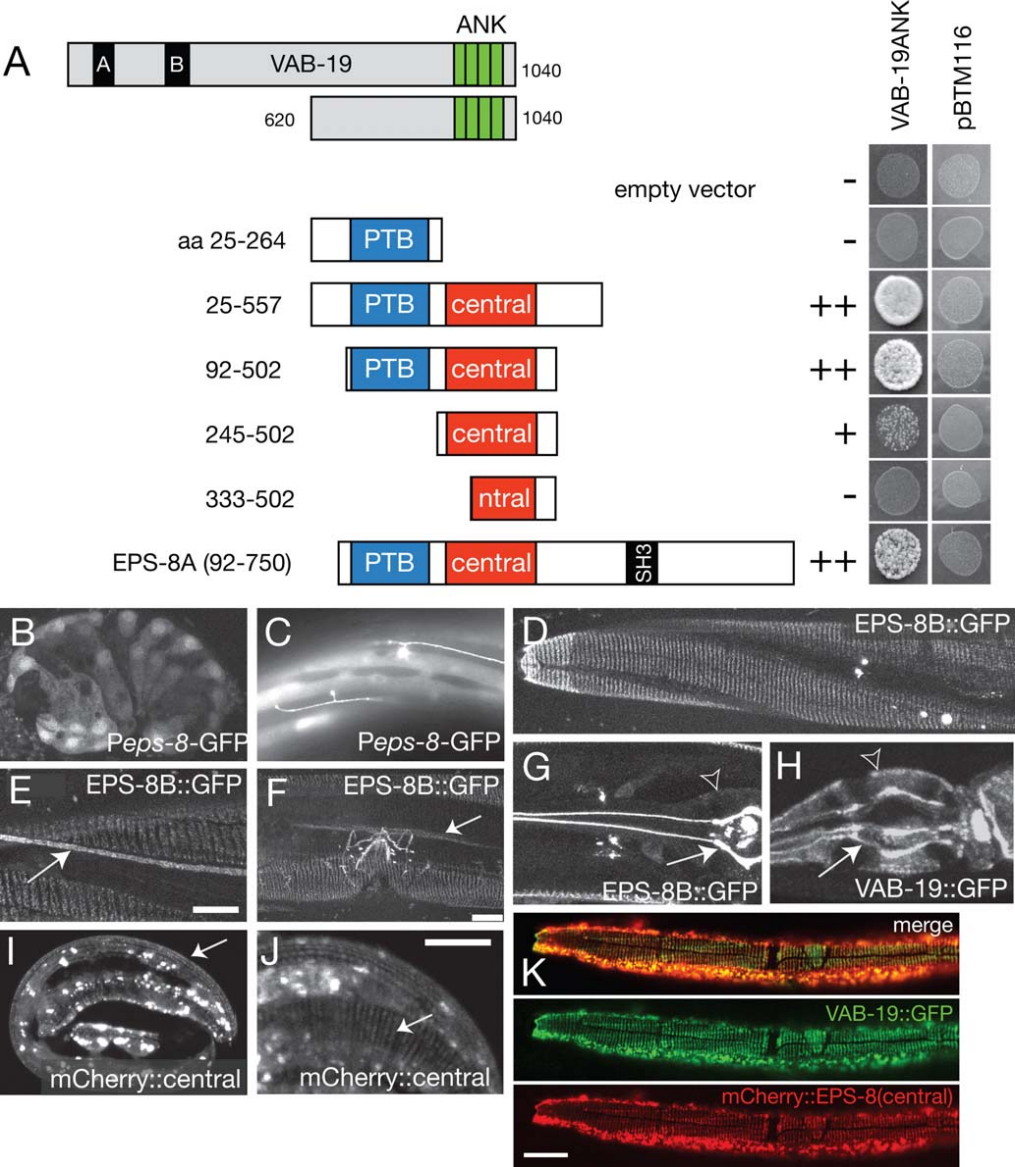
EPS-8 interacts with VAB-19 and colocalizes with VAB-19 at epithelial attachment structures. (A) The VAB-19 ANK repeat domain interacts with the central domain of EPS-8. The initial interacting clone corresponds to almost full length EPS-8A. Deletion derivatives of EPS-8A were screened for interaction with VAB-19ANK in yeast. A fragment containing the central domain (EPS-8A residues 245–502) is sufficient to interact with VAB-19 ANK domains, although constructs containing part of the PTB domain interact more strongly. The PTB domain alone (residues 25–264) does not interact with VAB-19ANK. None of the constructs cause self-activation (pBTM116 controls). (B, C) GFP expressed under the control of a 3.3 kb *eps-8* promoter (P*eps-8-*GFP, *juEx526*) is first seen in ventral and dorsal epidermal cells at comma stage (350 min) and persists in these tissues throughout larval and adult stages. P*eps-8*-GFP was also expressed in sublateral neurons (arrow, C). The 3.3 kb *eps-8* promoter likely drives a subset of the *eps-8* expression pattern, as *eps-8* has been shown to be expressed in intestinal and pharyngeal cells [17]. (D) EPS-8B::GFP (*juEx700*) was localized to circumferential bands in muscle-adjacent epidermis in embryos (not shown) and in larvae. We detected EPS-8B::GFP at other known sites of trans-epidermal attachments: adjacent to mechanosensory neuron processes (arrow, E), and at the uterine seam cell attachment (arrow, F). EPS-8::GFP was also expressed in the uterus and in the excretory cell (not shown). (G) In pharyngeal marginal cells, P*vab-19-*EPS-8B::GFP was localized to the apical (lumenal) ends of attachment structures (arrow) and not to the basal surface (arrowhead), in contrast to VAB-19::GFP (*juEx433*), which is found at both basal and apical ends of pharyngeal marginal cells (arrowhead and arrow, respectively, H). The subcellular localization of EPS-8A::GFP in these tissues was indistinguishable from EPS-8B::GFP (not shown). (I, J) Localization of mCherry:: EPS-8(central) (*juEx1784*) to embryonic attachment structures (arrow). Truncated mCherry::EPS-8 fusion proteins also displayed aggregation (bright blobs); as these aggregates are not observed with GFP-tagged full length EPS-8 they may result from overexpression of truncated proteins. (K) Co-localization of P*dpy-7*-mCherry::EPS-8(central) (*juEx1784*) and VAB-19::GFP (*juEx433*) to attachment structures in larval epidermis. Scales, 10 µm.

To understand how VAB-19 regulates epidermal morphogenesis, we used the C-terminal ankyrin repeat domain of VAB-19 as bait in a yeast two-hybrid screen, and identified *C. elegans* EPS-8 as a VAB-19 interactor (see Materials and Methods). Eps8 proteins typically contain an N-terminal phosphotyrosine-binding (PTB) domain, a central domain conserved among the Eps8 family and implicated in EGFR binding, an SH3 domain, and a C-terminal ‘effector’ domain that binds actin [19]–[22]. We mapped the interacting domains between VAB-19 and EPS-8 in yeast (Figure 1A) and found that the central domain of EPS-8 (residues 272–502) was necessary for binding VAB-19. Constructs containing only the central domain (residues 245–502) displayed a weaker interaction with VAB-19ANK, suggesting that although the central domain is sufficient for an interaction, the combination of the PTB and central domains may be required for a strong interaction.

**Figure 2:**
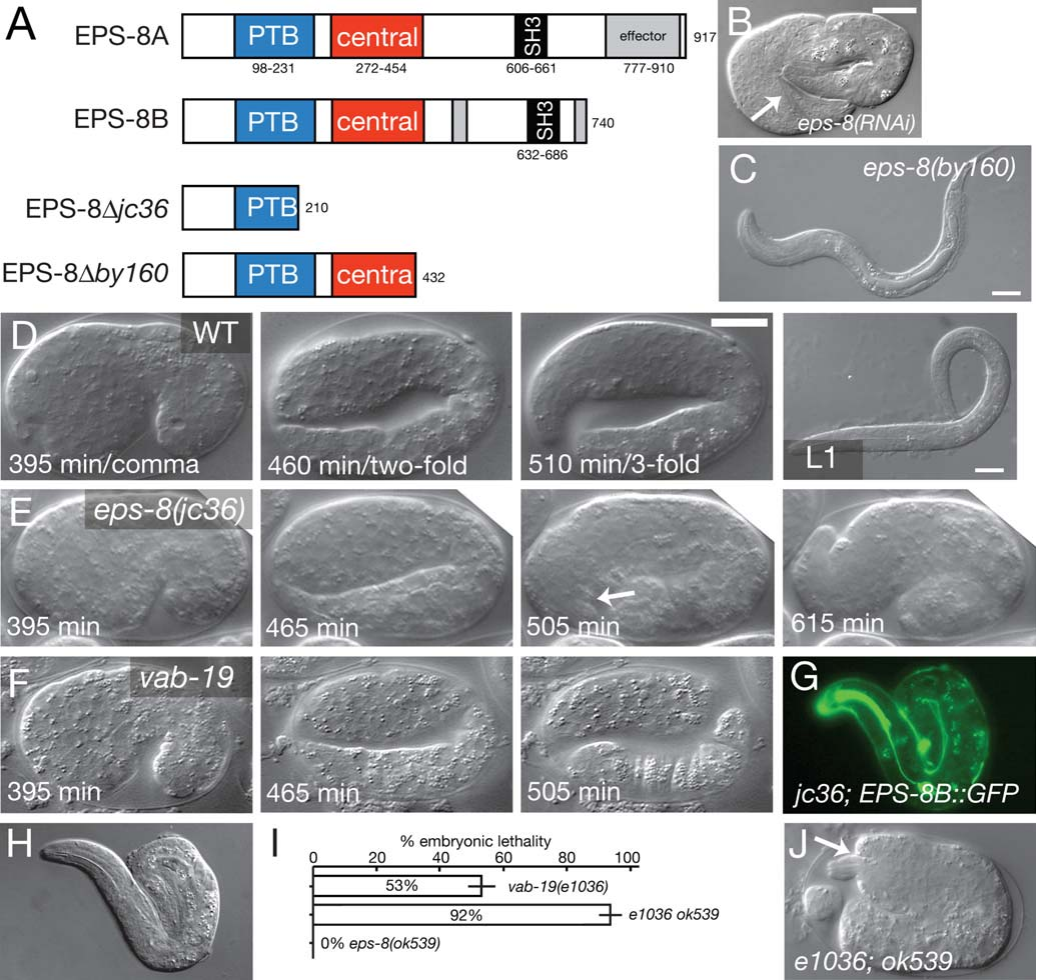
*eps-8* null mutants are defective in embryonic morphogenesis and muscle attachment. (A) Structure of EPS-8 isoforms and predicted effects of *by160* and *jc36* deletions. The location of the *ok539* intron deletion is shown in Croce et al., 2004 [17]. (B) Three-fold arrest with detached body wall muscles (arrow) in *eps-8(RNAi)* animal. (C) Arrested *eps-8(by160)* larva with normal epidermal morphology. (D–F) Frames from 4D time lapse movies of wild type (D), *eps-8(jc36)* mutants (E), and *vab-19(ju406)/Df* embryos (F) at time points equivalent to wild-type comma stage (395 minutes after first cleavage), two-fold (∼450 minutes), and three-fold (∼500 minutes). Like *vab-19* embryos, *eps-8(jc36)* mutants elongate at normal rates to the two-fold stage (n = 12 embryos recorded). *vab-19* embryos stop elongating within 5–10 minutes of the twofold stage, whereas all *eps-8(jc36)* embryos elongate to a 2.5- to 3-fold stage, stop elongating about an hour after the twofold stage, and then partly retract. *eps-8(jc36)* embryos show normal muscle twitching and, unlike *vab-19,* show vigorous movements within the eggshell; these movements stop after elongation arrest. Unlike *vab-19* mutants, which typically hatch as lumpy two-fold embryos, most *jc36* embryos do not hatch. Scales, 10 µm. (G, H) Rescue of *eps-8(jc36)* elongation defects by P*vab-19*-EPS-8B::GFP (*juEx703*); GFP (G) and DIC (H). (I) Enhancement of *vab-19(e1036cs)* embryonic lethality by the *eps-8b(ok539)* allele; bars show mean±SEM for 5 complete broods for each genotype; P<0.001 by two-tailed t test. (J) Typical *vab-19(e1036) eps-8(ok539)* embryo from parents raised at 15°C, showing two-fold arrest and deformed head epidermis (arrow).

*vab-19* is expressed in dorsal and ventral epidermal cells from early elongation stages onwards [11]. We found that *eps-8* transcriptional reporters were likewise expressed in dorsal and ventral embryonic epidermal cells beginning soon after epidermal enclosure (Figure 1B) and persisting in larval and adult stages (Figure 1C). To examine subcellular localization of EPS-8 within epidermal cells we inserted GFP into the N-terminus of EPS-8 and expressed it under the control of the epidermal-specific *vab-19* promoter. The *eps-8* locus generates two isoforms that differ in their C-termini, as a result of alternative splicing: EPS-8A contains the C-terminal actin binding ‘effector’ domain, whereas EPS-8B does not [17] (Figure 2A). Both EPS-8::GFP fusion proteins displayed indistinguishable patterns in the epidermis, and are referred to generically as EPS-8::GFP. In post-embryonic stages EPS-8::GFP was localized to circumferential bands in epidermis adjacent to muscles (Figure 1D), a pattern corresponding to trans-epidermal attachments. We also found EPS-8::GFP localized to other sites of trans-epidermal attachments (Figure 1E, F). These results indicate that in epidermal cells EPS-8, like VAB-19, is localized to sites of cell-matrix attachments.

To determine whether EPS-8 localizes to a specific part of cell-matrix attachments we examined attachment structures of pharyngeal marginal cells. Pharyngeal attachment structures resemble epidermal attachment structures in molecular composition, as they contain MH4-positive intermediate filaments, Plectin/VAB-10A, and Myotactin at their basal ends. Within these larger structures it is possible to distinguish apical versus basal localization of attachment structure components. EPS-8A::GFP and EPS-8B::GFP were enriched only at the apical ends of marginal cells (Figure 1G, arrow; basal surface of pharynx indicated by open arrowhead), in contrast to VAB-19::GFP, which is found at both apical and basal ends (Fig 1H, arrow and arrowhead respectively). Extrapolating from these data, EPS-8 may also be restricted to the apical ends of attachment structures in epidermal cells.

To determine whether the same regions that interact with VAB-19 are also important for localization we tested whether specific domains of EPS-8 could direct protein localization. We found that mCherry::EPS-8(central) localized to epidermal attachment structures (Figure 1I, J) whereas mCherry::EPS-8(PTB) did not (not shown). mCherry::EPS-8(central) colocalized with VAB-19::GFP at epidermal attachment structures (Figure 1K). VAB-19::GFP also displayed colocalization with endogenous EPS-8, as detected using the antibody K49 [17] (data not shown). The subcellular colocalization of EPS-8(central)::mCherry and VAB-19::GFP to epidermal attachment structures is consistent with a direct interaction of the EPS-8 central domain and VAB-19 *in vivo.*

### Loss of EPS-8 function results in epidermal elongation defects resembling those of *vab-19* mutants

Loss of *vab-19* function causes a distinctive combination of late epidermal elongation defects and detachment of body muscles, a syndrome often reflective of defects in muscle-epidermal adhesion or defects in epidermal attachment structures. RNA interference of *eps-8* caused epidermal morphogenesis phenotypes resembling those of *vab-19* mutants (Figure 2B). Injection of *eps-8* dsRNA into the syncytial gonads of wild type N2 hermaphrodites caused 100% lethality of F_1_ progeny laid between 5 and 20 h post injection (n = 569 progeny of 14 parents); of these progeny, 65.4% arrested at the twofold stage of elongation and displayed detachment of body muscles. The remainder of the *eps-8(RNAi)* progeny arrested as hatched threefold-stage embryos (e.g. Figure 2B) or L1s. This highly penetrant embryonic morphogenetic defect contrasts with the phenotypes of *eps-8(by160)* mutants, all of which display normal elongation and arrest as starved L1s (Figure 2C). To understand the basis of this discrepancy between these RNAi phenotypes and the *eps-8(by160)* phenotypes, we generated a new deletion mutation, *eps-8(jc36)* (Figure 2A; Methods). *eps-8(jc36)* mutants displayed 100% embryonic lethality due to fully penetrant defects in embryonic elongation and muscle attachment, corresponding to the most severe phenotypes observed in our RNAi experiments. Using timelapse analysis (n = 12) we found *eps-8(jc36)* embryos arrest during late elongation, although the stage of arrest is significantly later than that of *vab-19* mutants (Figure 2D–F). We conclude that while *eps-8* is essential for morphogenetic cell shape changes of embryonic epidermal elongation, it may act later in this process than does VAB-19.

The *jc36* deletion truncates EPS-8 isoforms within the PTB domain. In contrast, *by160*, which truncates EPS-8 in the central domain and deletes the SH3 domain of both isoforms, has no effect on epidermal morphogenesis, suggesting that the embryonic roles of EPS-8 do not require the SH3 domain or the C-terminal part of the central domain. A similar larval arrest is observed when the EPS-8A isoform is specifically targeted by RNAi [17]. A deletion of the 3′ UTR of the B isoform, *eps-8(ok539)*, eliminates expression of EPS-8B and has no effect on embryogenesis or larval morphogenesis (Ref. 17; Figure 2H). These allelic differences imply that the morphogenetic function of EPS-8 requires its PTB domain, and possibly the N-terminal part of the central domain, but not the SH3 domain or C-terminal actin binding domains. Further, the two EPS-8 isoforms may function redundantly in epidermal morphogenesis, as removal of either isoform alone does not affect morphogenesis. Consistent with this hypothesis, either EPS-8A::GFP or EPS-8B::GFP fusion proteins fully rescued *eps-8(jc36)* embryonic phenotypes when expressed under the control of the *vab-19* promoter (Figure 2G, H). These rescue experiments also indicate that EPS-8 functions cell autonomously in the epidermis to promote morphogenesis.

To test whether VAB-19 and EPS-8 display genetic interactions consistent with function in a common pathway we constructed double mutants using the partial loss of function allele *vab-19(e1036cs)* and the phenotypically silent allele *eps-8(ok539)*. *vab-19(e1036)* is a cold-sensitive allele that appears to eliminate function at 15°C [11]. Under conditions where *vab-19(e1036)* displayed 53% embryonic lethality, the *vab-19(e1036) eps-8(ok539)* double mutant displayed 92% embryonic lethality (Figure 2I) (P<0.001 by t test). Such *vab-19 eps-8* double mutants resembled the *vab-19* null phenotype, in that they arrested at the two-fold stage of embryogenesis with detached muscles and deformed head and tail epidermis (Figure 2J). However, at higher temperatures the penetrance of lethal phenotypes in *vab-19 eps-8* double mutants was not significantly different from *vab-19(e1036)* single mutants (not shown). These observations suggest that although elimination of the EPS-8B isoform does not itself have a dramatic effect on morphogenesis, it can affect development when VAB-19 is present in limiting amounts. This dose-dependent phenotypic enhancement is consistent with VAB-19 and EPS-8 acting in a common pathway or in closely related parallel pathways in controlling epidermal morphogenesis.

### EPS-8 is required for late aspects of attachment structure development and for normal actin organization

**Figure 3:**
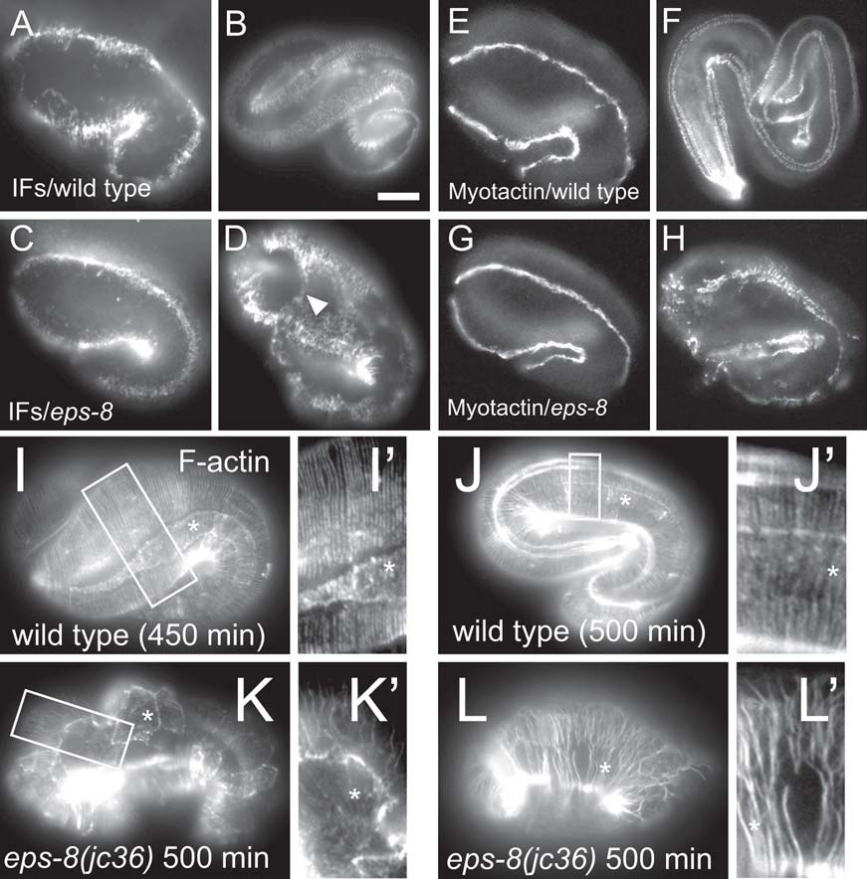
*eps-8* mutants display late-onset defects in epidermal attachment structure development and in the epidermal actin cytoskeleton. (A–D) Localization of epidermal intermediate filaments (MH4 immunostaining) in the wild type and in *eps-8(jc36)* mutants. At the 1.5-fold stage (A), IFs were restricted in muscle-adjacent region of epidermis. (B) After the twofold stage, IFs localize to regularly spaced circumferential stripes. (C–D) In *eps-8* mutants, IFs appear normal until after the twofold stage, when they expand into regions of epidermal cells that are not adjacent to muscle (arrowhead), compared to the wild type. (E–H) Myotactin expression (MH46 immunostaining) in the wild type and in *eps-8* mutants. (E, G) During early elongation (1.5 to two-fold), Myotactin appears normal in *eps-8* mutants. By the threefold stage, Myotactin localization refines to circumferential stripes in muscle-adjacent regions of epidermis (F). (H) *In eps-8* mutants, Myotactin is still localized to muscle-adjacent regions but remains in longitudinal bands rather than circumferential stripes. (I, J) In the wild type, circumferential bundles of F-actin (phalloidin staining) are continuous around the embryo. Insets I' and J' show actin bundles at higher magnification. In *eps-8* mutants, actin filaments were more randomly oriented or missing from the apical surface of the epidermis (K, K'). Actin filaments were also disorganized and fragmented in lateral epidermal cells (L, L', asterisk). Scale, 10 µm.

As EPS-8 appears to be a new component of epidermal attachment structures, we examined the effect of *eps-8* mutations on other attachment structure components. During early and intermediate (two-fold) stages of epidermal elongation, the localization of intermediate filaments (IFs) was normal in *eps-8* mutants (Figure 3A, C). However, during later elongation, IFs became delocalized, expanding into regions not adjacent to body wall muscle with slightly altered patterns of circumferential bands (compare Figure 3B and D). We conclude that EPS-8 is not essential for the initial assembly of attachment structures. We also examined the localization of the receptor-like protein Myotactin, which normally becomes refined at the two-fold stage from broad longitudinal bands adjacent to muscle into circumferential stripes corresponding to attachment structures [24], [25]. The early localization pattern of Myotactin into longitudinal bands was normal in *eps-8* embryos (Figure 3E, G). However, Myotactin never reorganized into circumferential stripes (compare Figure 3F and H). These effects on IFs and Myotactin in *eps-8* mutant embryos closely resemble those of *vab-19* mutants [11]. Expression of another cell junction marker, the apical junctional marker AJM-1 [23] in *eps-8(jc36)* mutants was similar to wild type (not shown), indicating that epidermal cells are specified correctly and have normal lateral cell–cell contacts.

Because *eps-8* mutants have defects in elongation, we tested whether EPS-8 is required for normal epidermal actin distribution. During elongation, actin normally becomes circumferentially oriented in apical bundles (CFBs) in the epidermis (Figure 3I,J). Actin distribution in *eps-8* mutants became disorganized as the mutant embryos developed to the two-fold stage. Actin CFBs were often mis-oriented or splayed (Figure 3K). Interestingly, disorganization of actin bundles was often most severe in the lateral epidermis or seam (Figure 3K, L; seam cells indicated by asterisks), where actin filaments were pulled away from each other generating fragmented actin CFBs. Because EPS-8 is expressed only in ventral and dorsal epidermis, disorganization of actin bundles within the lateral epidermis may be a secondary effect of actin disorganization in dorsal and ventral cells.

### Evidence that VAB-19 recruits EPS-8 to attachment structures

**Figure 4:**
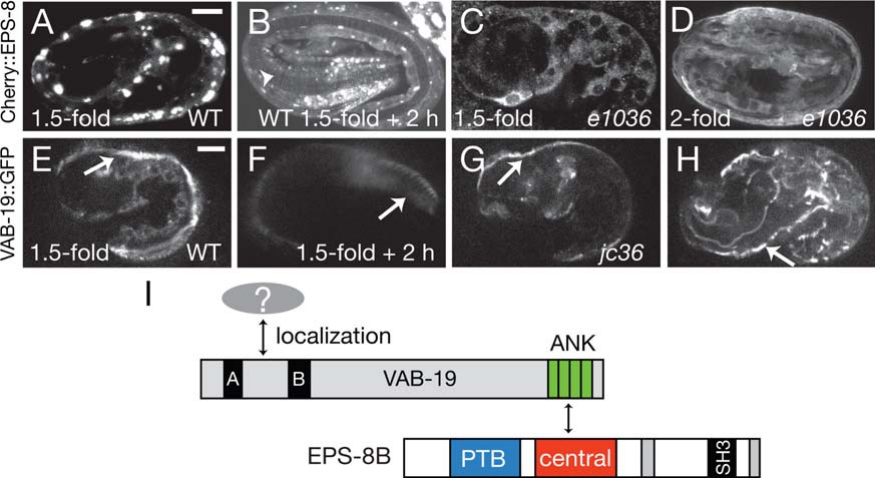
Molecular epistasis, order of recruitment, and model for interaction of VAB-19 and EPS-8. (A) In early elongation EPS-8 (mCherry::EPS-8(central), *juEx1784*) is either diffuse or aggregated and unlike VAB-19 does not localize to longitudinal bands adjacent to muscle (cf. panel E). By the threefold stage of elongation mCherry::EPS-8(central) is localized to circumferential stripes corresponding to attachment structures (arrowhead, B). (C, D) In *vab-19(e1036)* mutant embryos grown at 15°C, mCherry::EPS-8 is diffuse and does not localize to attachment structures at 1.5-fold (C) or two-fold (D). (E,F) In wild-type embryos VAB-19::GFP (*juIs169*) is localized to longitudinal bands at the 1.5-fold stage (arrow, E) and by the threefold stage becomes localized to circumferential stripes (arrow, F). The initial phase of VAB-19::GFP localization to longitudinal bands occurs in *eps-8(jc36)* mutants (arrow, G). However VAB-19::GFP never became clearly localized to circumferential stripes in later *jc36* embryos, remaining in longitudinal bands (arrow, H). (I) Model for the VAB-19/EPS-8 pathway. VAB-19 localization is directed by its N-terminal motifs, interacting with unknown components of attachment structures. The VAB-19 ANK repeats then recruit EPS-8 by interacting with its central domain. EPS-8 function may involve further interactions mediated by the EPS-8 PTB domain.

Although VAB-19 and EPS-8 both promote attachment structure development, two lines of evidence suggest VAB-19 may act earlier than EPS-8. First, the elongation defects of *vab-19* mutants become apparent slightly earlier than those of *eps-8* mutants. Second, whereas VAB-19 is localized to attachment structures beginning in early elongation (1.5-fold), EPS-8 fusion proteins did not appear clearly localized to attachment structures until the three-fold stage (Figure 4A, B). These differences in phenotype and localization suggest VAB-19 might act earlier than EPS-8 in a pathway of attachment structure biogenesis.

To further test this hypothesis we investigated whether VAB-19 was required for EPS-8 localization, or vice versa. In *vab-19(e1036)* mutants at the restrictive temperature mCherry::EPS-8(central) never became localized to circumferential stripes (Figure 4C,D), suggesting EPS-8 localization is dependent on VAB-19. Conversely, functional VAB-19::GFP was correctly localized to longitudinal bands in *eps-8(jc36)* embryos prior to their elongation arrest (Figure 4G), although after arrest VAB-19::GFP became disorganized (Figure 4H). These results indicate that VAB-19's initial localization to muscle-adjacent regions of the epidermis is independent of EPS-8.

## Discussion

We have identified a novel role for the cell signaling adaptor EPS-8 in *C. elegans* epithelial cell-matrix attachments. In other organisms Eps8 participates in several distinct processes via interactions mediated by its C-terminal SH3 or effector domains [17], [21], [26], [27]. However, our analysis indicates these known pathways are unlikely to account for the role of EPS-8 in the *C. elegans* embryonic epidermis, which we find is independent of the SH3 domain and effector domains. Instead, the N-terminal PTB and the central domain of EPS-8 are important for its role in cell-matrix attachments. Our data are consistent with a model (Figure 4M) in which VAB-19 is initially localized to cell-matrix attachments via motifs in its N-terminus. As yet it is unknown which protein(s) interact with the VAB-19 N-terminal motifs. A candidate is VAB-10A, which is present at all VAB-19-containing cell-matrix attachment structures [9]. Our attempts to test this possibility using the two-hybrid procedure have so far been inconclusive due to the strongly self-activating properties of the relevant VAB-19 and VAB-10 domains in LexA fusions (not shown). The VAB-19 ANK repeats then provide an interaction surface that recruits EPS-8 isoforms via the EPS-8 central domain. Localized EPS-8 might then interact with, recruit, or exclude additional proteins via its PTB domain, or VAB-19 and EPS-8 might together form a complex that recruits additional proteins. In vivo ligands for the Eps8 PTB domain have not been reported; screening of peptide libraries indicates the Eps8 PTB domain recognizes NPXY motifs independent of phosphotyrosine [28]. Finding additional interacting partners for Eps8 PTB domains could elucidate its function at cell matrix attachments.

Mammalian Eps8 binds the adaptor IRSp53, via Proline rich surfaces in the Eps8 N- and C-termini, forming a complex with actin-bundling activity [29], [30]. *C. elegans* EPS-8 lacks the N-terminal PXXP motif implicated in IRSp53 binding, and the *C. elegans* genome does not appear to encode an ortholog of IRSp53. Because VAB-19 and EPS-8 do not colocalize with the circumferential actin bundles in the epidermis, it is possible that the EPS-8/VAB-19 pathway functions differently from EPS-8/IRSp53 in the actin cytoskeleton.

Although as yet there is no evidence that this cell-matrix attachment role of Eps8 is conserved in other organisms, it is interesting to note that the Drosophila gene *arouser* encodes Eps8-related proteins that, like *C. elegans* EPS-8B, lack a C-terminal effector domain [16] (see alignment in Figure S1), and so presumably do not directly bind actin. The Aru and EPS-8B proteins also share identical C-termini (Figure S1), suggesting that this part of the protein may also be of functional significance. As Drosophila also encodes an ortholog of VAB-19, the EPS-8/VAB-19 interaction at cell matrix attachments could be phylogenetically conserved.

## Materials and Methods

### Strains and genetics

*C. elegans* was cultured as described [31]. Bristol N2 was used as the wild type. Mutations used in this study are: *eps-8(jc36)*, *eps-8(by160), eps-8(ok539),* and *vab-19(e1036*cs*)*.

*eps-8(jc36)* was isolated using standard methods [32] from an EMS mutagenized library of worms with the following primers: eps8ex4el ttgaagtgaatctaccgggc, eps8ex4er tggtatcgctccagatttcc, eps8ex4il ctagcaaagcccggtagatg, eps8ex4ir ctttcgaggtcgaaggaaga. *jc36* contains a 1538 bp deletion with breakpoints tctaaacattaa/aaagtgaaacggtcacag, extending from intron four to intron five and completely removing exon 5; *jc36* also contains a second deletion of 10 bp in intron 4. Removal of exon 5 is predicted to result in translation of the first 210 residues of EPS-8, followed by 23 missense residues and a premature stop codon. *eps-8(jc36)* was outcrossed to N2 six times before phenotypic analysis.

### EPS-8 molecular biology and transgenes

cDNAs for *eps-8* had been isolated in a genome-wide EST project (Y. Kohara). yk404f6 contains the full length EPS-8B cDNA. yk310f7 contains a partial EPS-8A cDNA lacking approximately the first 525 bases of the ORF. To construct a full length EPS-8A cDNA (pCZ613), the *Xba* I-*Nco* I cDNA fragment in yk310f7 was replaced with the corresponding fragment from yk404f6.

To examine activity of the *eps-8* promoter we amplified a 3.3 kb genomic fragment 5′ to the *eps-8* start codon and cloned it into the GFP vector pPD95.75. We injected these constructs using standard procedures [33]. All transgenic lines were by injection of 5-10 ng/µl of test DNA and 50 ng/µl pRF4 co-injection marker DNA. Three *Peps-8-*GFP transgenic lines (*juEx526-528*) displayed indistinguishable expression patterns.

To express EPS-8 isoforms in embryonic dorsal and ventral epidermal cells and in pharyngeal marginal cells we used the *vab-19* promoter. To make the P*vab-19*-driven EPS-8A::GFP construct, a 3.3 kb *Xba* I - *Bst*E II fragment of *vab-19* genomic DNA containing the promoter, first exon and part of the first intron (pCZ448) was inserted into pCZ613 digested with *Sma* I and *Not* I, creating pCZ614. GFP coding sequence from pPD47.52 (Fire lab vector kit) was inserted in-frame at the *Aat* II site of pCZ614, creating pCZ616. In these constructs GFP is inserted after codon 97 of EPS-8, immediately N-terminal to the PTB domain. Three transgenic lines (*juEx661, juEx662, juEx664*) were generated. The P*vab-19*-EPS-8B::GFP construct pCZ617 was generated in an equivalent strategy by inserting the 3.3 kb *vab-19* promoter sequence into yk404f6 and then subcloning GFP into the *Aat* II site. Four lines (*juEx700-703)* were obtained. Both epidermally expressed EPS-8A and EPS-8B transgenes fully rescued the elongation defects of *eps-8(jc36)* mutants; transgenic animals arrested in larval stages because the transgenes do not rescue the *eps-8* intestinal defects.

For the EPS-8 domain localization constructs we used Gateway recombination cloning (Invitrogen). We generated Gateway entry clones corresponding to the PTB and central domains (residues 1–185 and 186–580 of EPS-8A) and recombined these clones with a destination vector (pCZGY500) containing the epidermal specific *dpy-7* promoter [34] and a *C. elegans*-optimized version of mCherry [35] as an N-terminal tag. P*dpy-7*-mCherrry::EPS-8(PTB) (pCZGY595) and P*dpy-7*-mCherrry::EPS-8(central) (pCZGY594) DNAs were injected at 50 ng/µl with the coinjection marker P*ttx-3*-GFP to generate transgenic arrays *juEx1776-1781* and *juEx1782-1785* respectively.

### Yeast two-hybrid screen

The VAB-19 C-terminal ANK repeat domain (residues 620–1040) was amplified by PCR and cloned into the *Sal* I site of pBTM116, in-frame with the LexA DNA binding domain, creating pCZ618. A *C. elegans* embryonic cDNA library in the pACT2 vector (Clontech) was generously provided by Z. Zhou and H.R. Horvitz. DNA was transformed into the L40 yeast strain stably transformed with LexA-driven *HIS3* and LexA-driven *LacZ*. Transformants were selected for the ability to grow on -His plates supplemented with 2 mM 3-amino-1,2,4-triazole and subsequently screened for β-galactosidase synthesis. From a screen of 1.3×10^6^ clones we obtained 29 positive interacting clones. We tested the most strongly interacting genes defined by the clones for coexpression in epidermal cells using transcriptional reporters, and for phenotypic overlap with *vab-19* by RNA interference. To determine the regions of EPS-8 sufficient for interaction we used PCR to make deletion derivatives of the initial interacting clone and inserted these into the pACT2 vector using *Bam*H I and *Xho* I sites in the primers.

### RNA interference

The template for the sense and antisense *eps-8* RNA transcript was the *eps-8* cDNA subcloned into L4440 vector (clone MDC55). Sense and antisense RNAs were produced in separate transcription reactions using T7 primers and an RNA synthesis kit (Promega). Sense and antisense RNA were mixed to a final concentration of ∼1–5 µg/µl in water and injected into the gonad. We scored progeny broods laid by single injected animals at 20°C from 5–20 h post-injection. For 4-D imaging, embryos were obtained from L4 hermaphrodites injected with 4 µg/µl dsRNA generated from cDNA yk393c10 with Ambion MEGAScript T3 and T7 kits using standard methods.

### Time lapse imaging

To analyze the VAB-19::GFP or mCherry::EPS-8 in live embryos (images in Figure 4) we used either a Zeiss LSM510 confocal microscope or a spinning disk confocal microscope (Solamere Technology Group, Salt Lake City, UT), consisting of a Zeiss Axiovert 200 stand, a Yokogawa CSU-X1 high speed confocal scan head, a Photometrics Cascade II EMCCD camera, and appropriate laser lines (491 and 561 nm).

### Immunofluorescence and phalloidin staining

Whole mount immunofluorescence staining was performed following the Finney-Ruvkun protocol [36]. To analyze EPS-8::GFP in embryos, Clorox treated animals were fixed in MRWB containing 1% paraformaldehyde for 3.5 hrs on ice and incubated with anti-GFP antibodies (Chemicon) at 1∶100 dilution and appropriate secondary antibodies. MH4 and MH46 (Developmental Hybridoma Studies Bank, University of Iowa) were used at 1∶300 dilution. Images were collected using a Zeiss Axioskop or Zeiss LSM510 confocal. Phalloidin staining was performed as described [11]. Images presented in Figure 3 and 4 are representative of 5–10 embryos examined for each condition.

## Supporting Information

Figure S1 Similarity of C. elegans EPS-8B and Drosophila Arouser (A) Domain organization of C. elegans EPS-8B, Drosophila melanogaster Arouser isoform PA, and Human Eps8. PTB and SH3 domains are annotated using alignments with NCBI conserved domains, and the central domain is defined according to Tocchetti et al., 2003 [16]. (B) ClustalW 2.0 alignment of the EPS-8B central domain and N-terminus with that of Drosophila Aru and human Eps8 (excluding the effector domain). An earlier alignment of C. elegans EPS-8 in Tocchetti et al., 2003, was based on an inaccurate gene structure prediction; the correct EPS-8B sequence is used here. EPS-8B and Aru have identical C-termini: GKRGEFRYF.
(1.29 MB EPS)
